# Assessing the effect of hypoxia on cardiac metabolism using hyperpolarized ^13^C magnetic resonance spectroscopy

**DOI:** 10.1002/nbm.4099

**Published:** 2019-05-15

**Authors:** Lydia M. Le Page, Oliver J. Rider, Andrew J. Lewis, Victoria Noden, Matthew Kerr, Lucia Giles, Lucy J.A. Ambrose, Vicky Ball, Latt Mansor, Lisa C. Heather, Damian J. Tyler

**Affiliations:** ^1^ Department of Physiology, Anatomy and Genetics University of Oxford Oxford UK; ^2^ Department of Physical Therapy and Rehabilitation Science University of California San Francisco San Francisco USA; ^3^ Department of Radiology and Biomedical Imaging University of California San Francisco San Francisco USA; ^4^ Oxford Centre for Clinical Magnetic Resonance Research, Division of Cardiovascular Medicine University of Oxford Oxford UK

**Keywords:** cardiac metabolism, hyperpolarized ^13^C, hypoxia, magnetic resonance spectroscopy

## Abstract

Hypoxia plays a role in many diseases and can have a wide range of effects on cardiac metabolism depending on the extent of the hypoxic insult. Noninvasive imaging methods could shed valuable light on the metabolic effects of hypoxia on the heart *in vivo*. Hyperpolarized carbon‐13 magnetic resonance spectroscopy (HP ^13^C MRS) in particular is an exciting technique for imaging metabolism that could provide such information.

The aim of our work was, therefore, to establish whether hyperpolarized ^13^C MRS can be used to assess the *in vivo* heart's metabolism of pyruvate in response to systemic acute and chronic hypoxic exposure.

Groups of healthy male Wistar rats were exposed to either acute (30 minutes), 1 week or 3 weeks of hypoxia. *In vivo* MRS of hyperpolarized [1‐^13^C] pyruvate was carried out along with assessments of physiological parameters and ejection fraction. Hematocrit was elevated after 1 week and 3 weeks of hypoxia.

30 minutes of hypoxia resulted in a significant reduction in pyruvate dehydrogenase (PDH) flux, whereas 1 or 3 weeks of hypoxia resulted in a PDH flux that was not different to normoxic animals. Conversion of hyperpolarized [1‐^13^C] pyruvate into [1‐^13^C] lactate was elevated following acute hypoxia, suggestive of enhanced anaerobic glycolysis. Elevated HP pyruvate to lactate conversion was also seen at the one week timepoint, in concert with an increase in lactate dehydrogenase (LDH) expression. Following three weeks of hypoxic exposure, cardiac metabolism of pyruvate was comparable with that observed in normoxia.

We have successfully visualized the effects of systemic hypoxia on cardiac metabolism of pyruvate using hyperpolarized ^13^C MRS, with differences observed following 30 minutes and 1 week of hypoxia. This demonstrates the potential of *in vivo* hyperpolarized ^13^C MRS data for assessing the cardiometabolic effects of hypoxia in disease.

**Abbreviations used**
BOLD: blood‐oxygen‐level dependent
HIF: hypoxia‐inducible factor
HP: hyperpolarized
LDH: lactate dehydrogenase
PDH: pyruvate dehydrogenase
PDK: pyruvate dehydrogenase kinase
PET: positron emission tomography

## 1 INTRODUCTION

Oxygenation of tissue is key to survival and maintenance of organ health. The heart has the potential to be exposed to a spectrum of hypoxic insults, ranging from mild and transient, to prolonged and severe. The metabolic effects of acute hypoxia are well documented, and notably involve increased glycolytic flux and transient lactate acidosis.^1^, ^2^ Prolonged and severe hypoxia requires reprogramming of cardiac metabolism; the heart downregulates oxygen‐consuming processes and upregulates glycolysis in an attempt to maximize ATP production under oxygen‐restricted conditions.^3^, ^4^, ^5^ The effects of chronic hypoxia are observed in response to high altitude,^6^ or as a factor in many pathological conditions; examples include chronic obstructive pulmonary disease,^7^ complications in pregnancy,^8^ sleep apnoea,^9^ myocardial infarction (the peri‐infarct region)^10^ and heart failure.^11^

However, much of this existing literature relies on *ex vivo* assessment of the metabolic changes that occur. As such, noninvasive *in vivo* measures of the effect of oxygen levels on cardiac tissue would be valuable, especially as the hypoxic response can be very transient.^12^ Imaging techniques have begun to probe *in vivo* oxygen levels, and current prominent methods include blood‐oxygen‐level dependent (BOLD) MRI and positron emission tomography (PET) imaging, although neither is standard clinical practice as yet. BOLD MRI enables assessment of vascular oxygenation using the paramagnetic nature of deoxyhemoglobin to create image contrast^13^; this technique has not yet reached the clinic due to a combination of many challenges including low signal‐to‐noise and a need for robust analysis,^14^ which studies have begun to address.^15^ PET probes in development include ^18^F‐FAZA, which accumulates in the presence of low oxygen.^16^ This probe is a more recent development showing improved signal‐to‐noise over the more clinically studied ^18^F‐FMISO; both tracers have been studied with a focus on tumor imaging thus far. Further research will clarify their clinical potential in the field of cardiac imaging.

Spectroscopic imaging holds potential for providing noninvasive, nonradioactive metabolic data. Imaging of carbon‐13 (^13^C) in particular can be very informative given the abundance of carbon present in metabolites, including those in pathways affected by oxygen level. Although ^13^C spectroscopy suffers from inherently low sensitivity *in vivo*, the advent of hyperpolarized ^13^C magnetic resonance spectroscopy (HP ^13^C MRS) offers the unique ability to measure the rate of enzyme flux *in vivo*.^17^ It provides an enhancement of the ^13^C signal of >10 000‐fold and, as such, enables a noninvasive measurement of enzymatic flux in real time. In the heart, the glycolytic pathway is central to the metabolic changes that occur as oxygen levels fall. The most established hyperpolarized ^13^C‐labeled probe, [1‐^13^C] pyruvate, is relevant to this pathway, as it allows us to visualize the fate of pyruvate either through mitochondrial pyruvate dehydrogenase (PDH) to bicarbonate, or through cytosolic lactate dehydrogenase (LDH) into lactate.^18^ A previous study by Laustsen et al^19^ showed the value of hyperpolarized pyruvate in the investigation of hypoxia, in the diabetic rat kidney, demonstrating an ability to measure increased lactate production after 15 minutes of hypoxic anaesthesia. Hypoxia is also one of many pathological factors of tumor development,^20^ fluctuating over time and in regions of the tumor,^21^ and as such Iversen et al used HP^13^C MRS in a mouse tumor model, showing that inspiration of a hypoxic atmosphere caused increased lactate production in tumors.^22^ Oxidative stress has been investigated in a few noncardiac studies, using HP dehydroascorbate,^23^, ^24^ but the toxicity of this compound may limit translation to clinical studies.^25^ The challenges and future of hyperpolarized probes for assessing renal and cardiac oxygen metabolism have been discussed in a review by Schroeder and Laustsen;^26^ thus far, no studies have investigated the use of HP ^13^C MRS to assess the effect of hypoxia on pyruvate metabolism in the *in vivo* heart.

**Figure 1:**
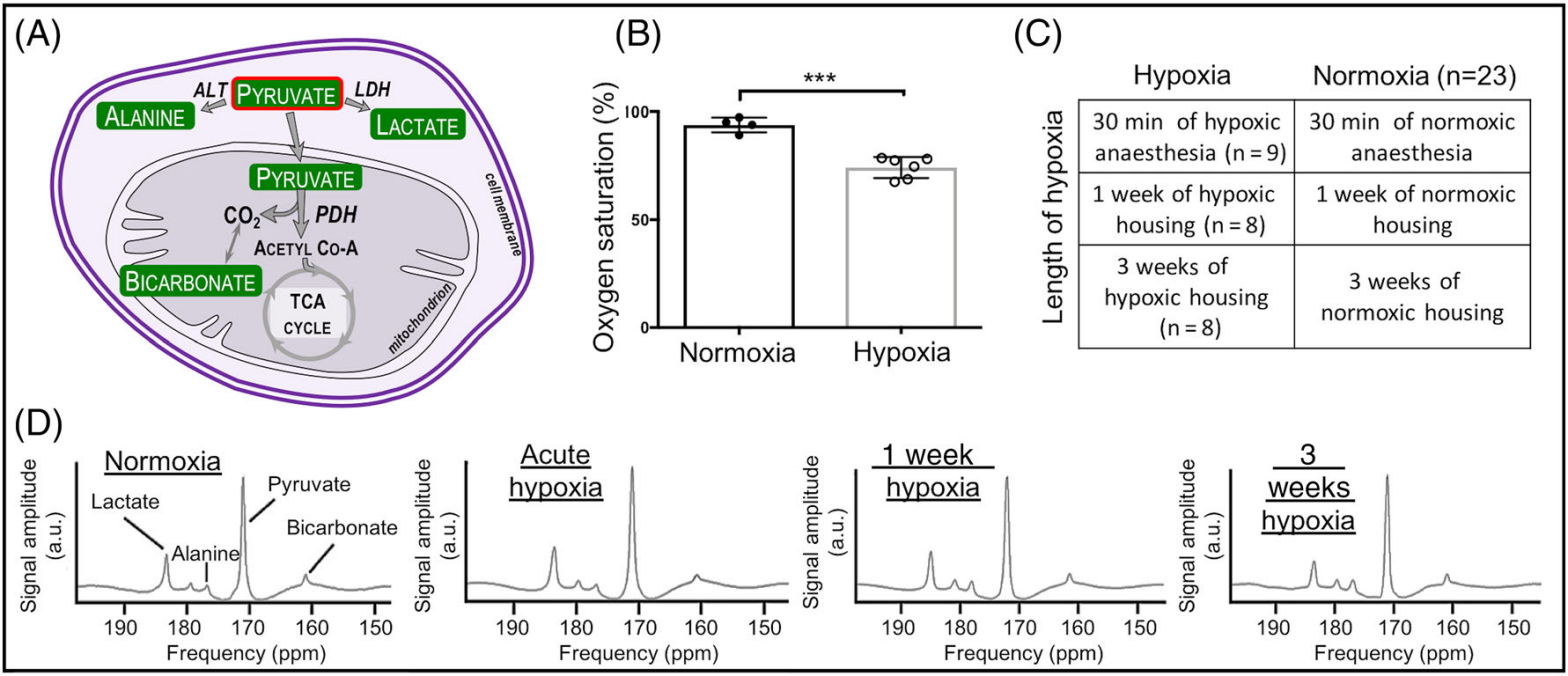
(A) Biochemical pathways visualized using HP [1‐^13^C] pyruvate (outlined in red). (B) Oxygen saturation of animals housed in the hypoxic chamber; ***p = 0.0001. (C) Experimental animal groups for three lengths of hypoxic exposure. Normoxic data subsequently treated as one group, n = 23. (D) Example summed spectra from each timepoint

In this study, we have therefore assessed the effect of three lengths of hypoxic exposure—30 minutes, 1 week and 3 weeks—on the *in vivo* rat heart, using hyperpolarized [1‐^13^C] pyruvate. We have measured the conversion of HP pyruvate to bicarbonate, lactate and alanine (Figure 1A shows the biochemical pathways involved). The level of oxygen saturation in the blood was matched across groups, and established following measurement in animals housed at 11% oxygen from previous rodent studies in our laboratory.^3^, ^4^ Alongside cardiac metabolism by MRS, we assessed ejection fraction by CINE MRimaging, and measured heart rate and respiration rate in all groups. We further measured body weight and hematocrit in the longer exposure groups (one week and three weeks hypoxia). In these latter groups, expression levels of cardiac PDH regulators pyruvate dehydrogenase kinase (PDK) 1, 2 and 4, and the expression level of lactate dehydrogenase (LDH), responsible for conversion of pyruvate to lactate, were also measured in cardiac tissue.

## 2 METHODS

### 2.1 Animal handling

Male Wistar rats (initial body weight ~200 g, Harlan, UK) were housed on a 12:12 hour light/dark cycle in animal facilities at the University of Oxford. All imaging studies were performed between 06:00 am and 01:00 pm with animals in the fed state. All procedures conformed to the Home Office Guidance on the Operation of the Animals (Scientific Procedures) Act of 1986 and to University of Oxford institutional guidelines.

### 2.2 Hypoxic exposure

A group of hypoxically housed animals (*n* = 6) and a group of animals housed in normoxia (*n* = 4) were used to assess blood oxygen saturation. Saturation was measured to be 74 ± 2% (Figure 1B) in hypoxia, using a pulse oximeter on their hind paw (MouseOx, Starr Life Sciences, PA, USA). This concentration was subsequently matched for all hypoxic exposures.

### 2.3 Experimental groups for *in vivo* imaging

Three separate groups of animals were exposed to three lengths of hypoxia. Further control groups of animals experienced normoxia only. The groups are summarized in Figure 1C.

#### 2.3.1 30 minutes (acute) hypoxia

Animals (*n* = 9) were anaesthetized using isoflurane (2%) in 100% O_2_ (2L/min). Metabolic and functional data were acquired in normoxia as described in the imaging protocol below. Animals were then slowly introduced to hypoxia by increasing replacement of oxygen with nitrogen over 30 minutes, until a blood oxygen saturation which matched that of the animals housed in the hypoxic chamber was achieved (described above). A second injection of hyperpolarized [1‐^13^C] pyruvate was administered and a second data set acquired. Acute hypoxia elicited some rapid physiological responses such as increased ventilation and heart rate,^27^ which settled prior to data acquisition, allowing acquisition of data in a stable hypoxic state.

#### 2.3.2 1 week of hypoxia

Animals (*n* = 10) were housed in a normobaric hypoxic chamber for one week, during which time the oxygen concentration was reduced daily by 1–2% until on the final day the concentration was 11%. Animals were weighed daily, which resulted in brief exposure to normoxia (no longer than five minutes). Animals were subsequently anesthetized under hypoxia (O_2_/N_2_ mix) outside the chamber, before being placed in the MR system and then the imaging protocol was executed. A control group (*n* = 6) was housed outside the hypoxic chamber in room air (21% oxygen) for one week, from which normoxic data were acquired, under normoxic anesthesia.

#### 2.3.3 3 weeks of hypoxia

Animals (*n* = 8) were introduced to the normobaric hypoxic chamber as for the one week experiments, but remained in the chamber for a further 14 days at 11% oxygen. Animals were then anaesthetized under hypoxia outside the chamber (O_2_/N_2_ mix) and underwent the MR protocol as for the one week animals to obtain *in vivo* cardiac metabolic data. A control group (*n* = 8) was housed outside the hypoxic chamber in room air (21% oxygen) for three weeks, from which normoxic data were acquired, under normoxic anaesthesia.

### 2.4 Magnetic resonance (MR) protocol

Animals were anesthetized with isoflurane (3.5% induction and 2% maintenance), and subsequently maintained under normoxic or hypoxic anesthesia, as appropriate. Rats were positioned in a 7 T horizontal bore MR scanner interfaced to a Direct Drive console (Varian Medical Systems, Yarnton, UK), and a home‐built ^1^H/^13^C butterfly coil (loop diameter, 2 cm) was placed over the chest. Correct positioning was confirmed by the acquisition of an axial proton fast low‐angle shot (FLASH) image (TE/TR, 1.17/2.33 ms; matrix size, 64 x 64; FOV, 60 x 60 mm; slice thickness, 2.5 mm; excitation flip angle, 15°). An ECG‐gated axial CINE image was obtained (slice thickness: 1.6 mm, matrix size: 128 × 128, TE/TR: 1.67/4.6 ms, flip angle: 15°) at the level of the papillary muscles for ejection fraction calculation. An ECG‐gated shim was used to reduce the proton linewidth to ~120 Hz. Hyperpolarized [1‐^13^C] pyruvate (Sigma‐Aldrich, Gillingham, UK) was prepared by 40 minutes of hyperpolarization at ~1 K (prototype polarizer, 3.35 T) as described by Ardenkjaer‐Larsen et al,^17^ before being rapidly dissolved in a pressurized and heated alkaline solution. This produced a solution of 80mM hyperpolarized sodium [1‐^13^C] pyruvate at physiological temperature and pH, with a polarization of ~30%. One millilitre of this solution was injected over 10 seconds via a tail vein cannula (dose of ~0.32 mmol/kg). Sixty individual ECG‐gated ^13^C MR slice selective, pulse‐acquire cardiac spectra were acquired over 60 seconds after injection (TR, 1 s; excitation flip angle, 5°; slice thickness, 10 mm; sweep width, 13 593 Hz; acquired points, 2048; frequency centered on the C1 pyruvate resonance).^28^

### 2.5 Tissue collection

All animals were sacrificed with an overdose of isoflurane following completion of the MR protocol. The heart was rapidly removed, washed briefly in phosphate buffered saline, and snap‐frozen in liquid nitrogen.

### 2.6 Blood analyses

Samples of blood were collected from the chest cavity on sacrificing, and centrifuged at 8000 rpm for 10 minutes. Hematocrit was measured using a microhematocrit reader (Hawksley, UK).

### 2.7 Tissue analysis

For Western blotting of cardiac tissue from the 1 week and 3 week groups, frozen tissue was crushed and lysis buffer added before tissue was homogenized; a protein assay established the protein concentration of each lysate. The same concentration of protein from each sample was loaded onto 12.5% SDS‐PAGE gels and was separated by electrophoresis.^29^ Primary antibodies for PDK 1 and 2 were purchased from New England Biolabs and Abgent, respectively; an antibody for PDK4 was kindly donated by Prof. Mary Sugden (Queen Mary's, University of London, UK). A primary antibody for LDH was purchased from Abcam (ab52488). Even protein loading and transfer were confirmed by Ponceau staining (0.1% *w*/*v* in 5% *v*/v acetic acid, Sigma‐Aldrich), and internal standards were used to ensure homogeneity between samples and gels. Bands were quantified using UN‐SCAN‐IT gel software (Silk Scientific, USA) and all samples were run in duplicate on separate gels to confirm results.

### 2.8 Magnetic resonance data analysis

All cardiac ^13^C spectra were analyzed using the AMARES algorithm in the jMRUI software package.^30^ Figure 1D shows example spectra summed over 30 seconds of acquisition in normoxic animals, acutely hypoxic animals and animals housed in hypoxia for 1 and 3 weeks, showing cardiometabolic conversion of the injected hyperpolarized pyruvate into the downstream products, lactate, alanine and bicarbonate. Spectra were DC offset‐corrected based on the last half of acquired points. The peak areas of [1‐^13^C] pyruvate, [1‐^13^C] lactate, [1‐^13^C] alanine and [^13^C] bicarbonate at each timepoint were quantified and used as input data for a kinetic model based on that developed by Zierhut et al^31^ and Atherton et al.^32^ PDH flux was quantified as the rate of ^13^C label transfer from pyruvate to bicarbonate. The rate of ^13^C label transfer from pyruvate to lactate and alanine was used as a marker of lactate dehydrogenase activity and alanine aminotransferase activity, respectively. CINE images were analyzed using cmr42 software (Circle Cardiovascular Imaging, Calgary, Canada) by an experienced analyst blinded to experimental group.

### 2.9 Statistical analyses

No significant differences were observed between the three normoxic control groups (data acquired for a control group at each time point: acute, 1 week and 3 weeks) for any parameter; therefore, all normoxic values were combined for subsequent analysis. Values are reported as the mean ± standard deviation. Differences between groups were assessed using a one‐way ANOVA followed by a Tukey's multiple comparisons test. This was performed using GraphPad Prism version 6.0 for Mac OS X (GraphPad Software, La Jolla, CA, USA; www.graphpad.com). Statistical significance was considered if *p* ≤ 0.05.

## 3 RESULTS

**Figure 2:**
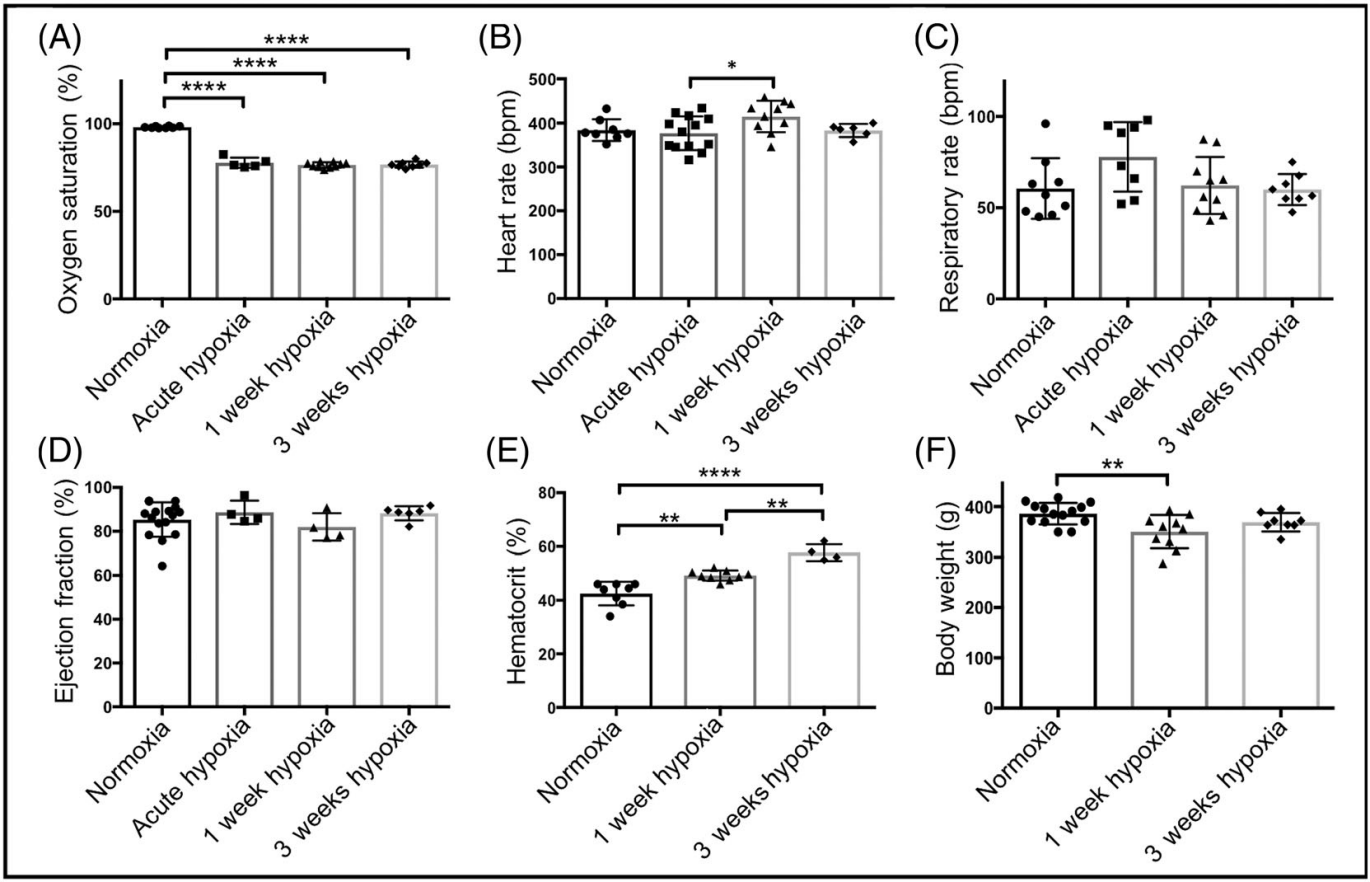
Effects of hypoxic exposure on (A) blood oxygen saturation, (B) heart rate, (C) respiration rate, (D) cardiac ejection fraction, (E) hematocrit levels and (F) body weight, all in comparison with normoxically housed animal data; *p ≤ 0.05; **p < 0.01; ****p < 0.0001

Oxygen saturation was successfully reduced in all hypoxic groups compared with normoxic data (Figure 2A).

### 3.1 Physiological effects of hypoxia

Hypoxia did not significantly affect respiration rate or left ventricular ejection fraction in any group (Figure 2B‐D). However, heart rate was significantly elevated in 1 week hypoxic animals compared with 30 minutes of hypoxia (*p* = 0.04). 1 week of hypoxia also caused a significant increase in hematocrit compared with normoxia (49.3 ± 0.6% and 43 ± 2%, respectively), and further, hematocrit in three week hypoxic animals was significantly increased compared with one week and normoxic values (58 ± 2%) (Figure 2E); this demonstrates systemic adaptation to hypoxia over time.

Animals housed in hypoxia for one week showed significantly lower body weights than normoxic animals. Following 3 weeks of hypoxia, however, body weights were no different from controls.

### 3.2 Metabolic effects of hypoxia

#### 3.2.1 *In vivo* data

**Figure 3:**
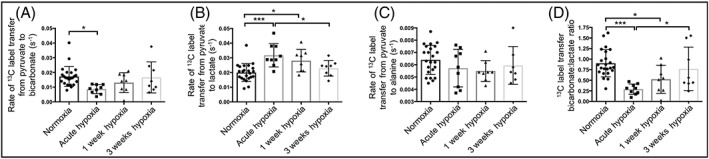
Following normoxia, 30 minutes, 1 week and 3 weeks of hypoxic exposure, rates of HP ^13^C label transfer from HP [1‐^13^C] pyruvate to (A) bicarbonate, (B) lactate and (C) alanine. (D) bicarbonate:lactate ratio for all timepoints; *p < 0.05; ***p < 0.001

Following 30 minutes of hypoxia, animals demonstrated a significant reduction in PDH flux (50%) compared with normoxic animals (0.009 ± 0.003 s^−1^ and 0.017 ± 0.007 s^−1^, respectively; Figure 3A). In contrast, both 1 and 3 weeks of hypoxic exposure did not show significantly altered PDH flux, with values not significantly different from controls (1 week hypoxia: 0.013 ± 0.007 s^−1^; 3 weeks hypoxia: 0.017 ± 0.011 s^−1^; normoxia: 0.017 ± 0.007 s^−1^).

A significant (58%) increase in HP ^13^C label transfer to lactate (Figure 3B) was observed when comparing 30 minutes hypoxic exposure with normoxic data (0.032 ± 0.008 s^−1^ and 0.020 ± 0.006 s^−1^, respectively), indicative of a short‐term metabolic shift towards anaerobic metabolism. After 1 week of hypoxia, the unchanged PDH flux was accompanied by an increased rate of ^13^C label transfer to lactate (by 40%) compared with normoxic animals (0.028 ± 0.008 s^−1^ and 0.020 ± 0.006 s^−1^, respectively). No difference in flux to ^13^C lactate was observed following 3 weeks of hypoxia compared with normoxic data (0.023 ± 0.002 s^−1^ and 0.020 ± 0.001 s^−1^, respectively). No change in the rate of ^13^C label transfer to alanine was seen at any timepoint (Figure 3C).

The bicarbonate:lactate ratio was significantly decreased in the acute hypoxic group compared with normoxia and 3 week hypoxic data (Figure 3D). Ratios following 1 week of hypoxia were also significantly lower than those observed in normoxia.

#### 3.2.2 Biochemical analyses

**Figure 4:**
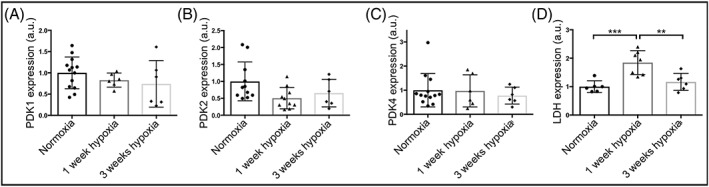
Western blot data from normoxic, one wk hypoxic and three wk hypoxic exposure showing protein expression levels of (A) PDK 1, (B) PDK2, (C) PDK4 and (D) LDH; **p < 0.01; ***p < 0.001

Cardiac tissue from the 1 week and 3 week hypoxic groups was assessed *ex vivo*. In agreement with the unchanged PDH flux at both these timepoints, no significant differences in the protein expression levels of the regulatory cardiac PDK isoforms (1, 2 and 4) were observed (Figure 4A‐C). A significantly higher expression of LDH was observed in the 1 week hypoxic tissue, in line with the increased HP pyruvate to lactate conversion seen *in vivo* (Figure 4D).

## 4 DISCUSSION

In hypoxia, metabolic changes have to occur in order for cardiac function to be maintained under the oxygen‐restricted conditions. Firstly, considering the response to acute hypoxia, the heart must rapidly shift metabolism towards a more anaerobic phenotype, which is characterized by increased glycolysis, increased lactate efflux^33^ and decreased oxidative mitochondrial metabolism. Indeed, in the animals exposed to 30 minutes of hypoxia, cardiac pyruvate to lactate conversion *in vivo* was significantly increased, PDH flux significantly decreased, and consequently bicarbonate: lactate ratios decreased. The rapid response that we observed, in line with the expected metabolic signature of anaerobic respiration, is likely mediated by changes in the NAD^+^/NADH ratio as a direct result of the decreased oxygen availability.^34^ The reduced oxygen results in decreased mitochondrial respiration,^4^ increasing NADH, inhibiting NAD‐dependent dehydrogenases such as PDH and promoting NADH‐dependent dehydrogenases such as LDH. In the context of disease, such measurements may be able to provide insight into the cardiometabolic effect of the transient hypoxic episodes that occur in sleep apnea, and the subsequent long‐term effects on cardiac pyruvate metabolism. Response to therapies could be similarly monitored using HP ^13^C MRS. This is particularly valuable given the increased risk of cardiovascular disease in those with sleep apnea.^35^

After 1 week of hypoxic exposure, we observed a significantly increased hematocrit level, as the animals underwent adaptation to the increasing level of hypoxia. This potentially indicates a partial adaptation to the hypoxic environment, a particularly viable suggestion when considered alongside the 3 week hematocrit data, which shows an additional significant increase in hematocrit. The increased hematocrit level demonstrated by our 1 week and 3 week hypoxic animals is a hallmark of systemic adaptation to physiological hypoxia, driven by HIF‐2α‐stimulated production of erythropoietin.^36^, ^37^ Our hypothesis of interim adaptation at 1 week of hypoxic exposure is supported by our observation of an increased heart rate ‐ a compensatory mechanism to ensure sufficient systemic oxygen delivery, and a significantly reduced body weight. Similar parameters have been observed in humans adapting to altitude showing increased heart rate^38^ and a lower calorie intake,^39^ the latter of which has been suggested to be due to increased leptin levels.^40^ Given the recent translation of HP technology to the clinical setting,^41^ metabolic changes observed here could now be confirmed in humans, either in those living at high altitude or who experience temporary exposure to hypoxia. Exact comparisons would need to take atmospheric pressure into consideration, given that our study was carried out using a normobaric hypoxic chamber.

Glycolytically‐ derived lactate was increased in the 1 week hypoxic animals, as assessed by HP pyruvate to lactate conversion, in line with significantly increased LDH expression in comparison with normoxic data. Bicarbonate:lactate was significantly decreased with respect to normoxic data (driven by the increased lactate), confirming that the metabolic balance between glycolysis and oxidative phosphorylation had not returned to normoxic levels at this timepoint. Glycolytic changes such as these have been reported to be predominantly HIF‐1α‐regulated,^42^ such as that of lactate dehydrogenase,^43^ the enzyme responsible for the HP conversion we measured *in vivo*. PDH flux was not decreased at one week, which was supported by our assessment of expression levels of its PDK regulators, perhaps unexpectedly due to previous studies discussing the hypoxia‐inducible nature of PDK1.^44^, ^45^ However, although studies such as those by Kim et al^44^ and Papandreou et al^45^ have shown upregulation of PDK1 in mouse embryonic fibroblasts following 24–72 hours in 0.5% hypoxia, a mouse study by Le Moine et al demonstrated no elevation of PDK1 expression in skeletal muscle following one week of hypoxic exposure.^46^ This latter study, alongside our data, demonstrates that conclusions drawn from cell studies do not necessarily translate *in vivo*.

Our 3 week hypoxic exposure resulted in no metabolic differences in the conversion of HP pyruvate to lactate or bicarbonate in comparison with normoxic data, as supported by measures of PDK and LDH expression. Previous studies from our group have shown that this 3 week protocol of chronic hypoxia at 11% oxygen is sufficient to metabolically reprogram the heart specifically to become more oxygen‐efficient.^5^ Further, studies in animal models of hypertrophy have revealed unchanged PDH activity^47^, ^48^ and no differences in PDK isoforms, which appeared at odds with cellular studies on hypoxia. Our data on healthy animals exposed to 3 weeks of hypoxia contribute to these observations and may in future help explain the situation in disease.

### 4.1 Limitations

This study did not measure *ex vivo* PDH activity, however, work by Atherton et al has demonstrated a significant correlation between *in vivo* data acquired using HP [1‐^13^C] pyruvate and PDH activity assessed from *ex vivo* tissue,^32^ and so we believe our data provide a valid measure of PDH activity.

A pulse‐acquire sequence was used in this study, and data were acquired using a surface coil. This is a limitation of this study given recent work by Wespi et al^49^ which demonstrated overestimation of cardiac lactate production with such a protocol. In our study, *ex vivo* analysis of LDH does support our *in vivo* conclusions, but future work will involve implementing more elegant acquisition protocols such as those described by Lau et al^50^ and Miller et al^51^ to provide more information on regional hypoxia within the heart and confirm our findings. Although HP ^13^C MRS alone is unlikely to provide a complete picture of hypoxic metabolism, this study shows its potential for providing a source of *in vivo* data.

Finally, normoxic animals were imaged using 100% oxygen, which, although a common procedure in preclinical animal studies, may exacerbate the differences we have seen here. Future studies could include anesthesia at a lower oxygen percentage.

## 5 CONCLUSION

In conclusion, we have demonstrated the ability of HP [1‐^13^C] pyruvate to noninvasively assess metabolic changes in the healthy heart in response to three lengths of exposure to hypoxia. This could, therefore, be a viable technique for assessing hypoxia in a wide range of diseases and in response to therapy.
